# Supplementary material for: Short Chain Fatty Acid Acetate Increases TNFα-Induced MCP-1 Production in Monocytic Cells via ACSL1/MAPK/NF-κB Axis
Source: Int J Mol Sci. 2021 Jul 19;22(14):7683. doi: 10.3390/ijms22147683 (PMC8304091; doi:10.3390/ijms22147683)
Supplement: Supplementary file 1 [file ijms-22-07683-s001.zip › ijms-1282253-supplementary.pdf]

Additional files/ Supplementary Figures

Title: Short chain fatty acid acetate increases TNFα-induced MCP-1 production in monocytic cells via ACSL1/MAPK/NF-kB axis

Authors: Areej Al-Roub, Nadeem Akhter, Amnah Al-Sayyar, Ajit Wilson, Reeby Thomas, Shihab Kochumon, Fatema Al-Rashed, Fahd Al-Mulla, Sardar Sindhu, Rasheed Ahmad

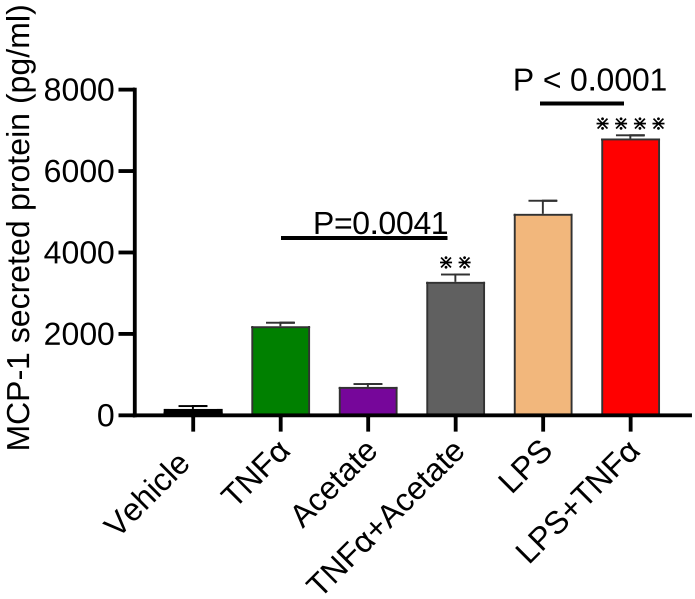

Figure S1. TNFα/LPS induces higher MCP-1 production compared to stimulation by TNFα/acetate. THP-1 monocytic cells were stimulated in triplicate wells with tumor necrosis factor (TNF)-α (10 ng/mL) with/without sodium acetate (100 mM) or TNFα (10 ng/mL) with/without lipopolysaccharide (LPS; 10 ng/mL) and cells were incubated at 37°C for 24h. Supernatants were harvested for determining MCP-1 levels by ELISA . The data obtained from 3 independent experiments with similar results show MCP-1 expression in response to co-stimulation by TNFα/acetate or by or TNFα/LPS. All data are expressed as mean ± SEM (n = 3).**$p < 0.01$, ****$p < 0.0001$.

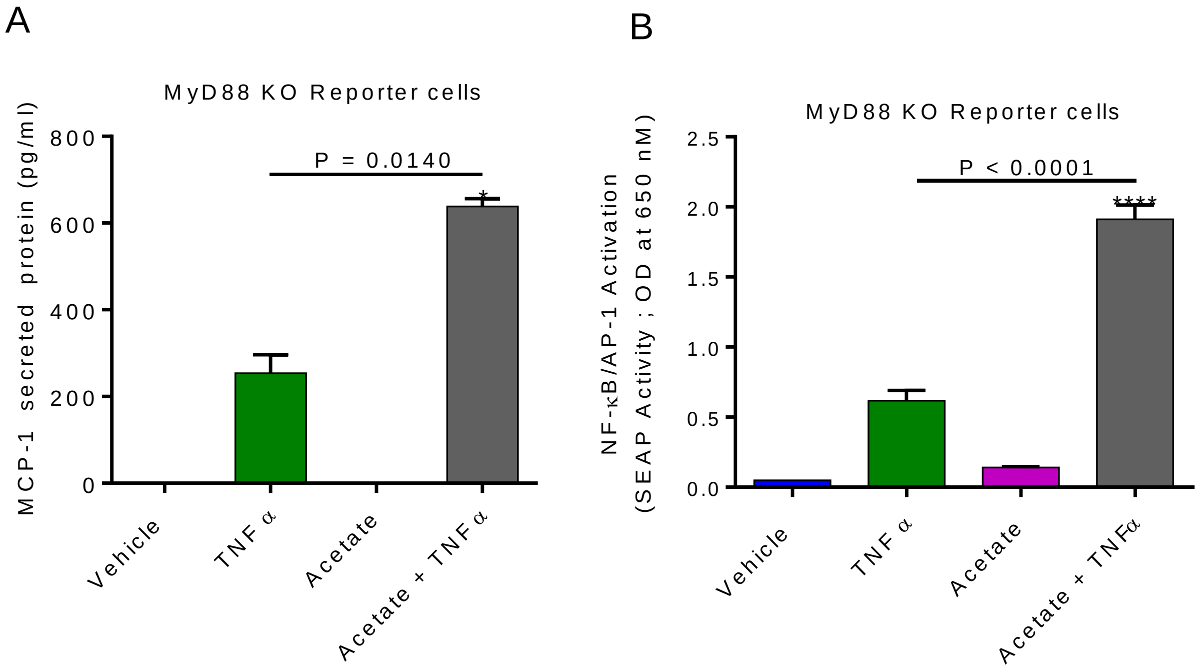

Figure S2. TNFα together with acetate increases MCP-1 and NF-κB/AP-1 activity independent of MyD88. (A) MyD88 Knockout reporter cells were treated as described. Culture media were collected. **(A)** MCP-1 protein was determined in the supernatant. (**B**)Cell culture media were assayed for SEAP reporter activity (degree of NF-κB/AP-1 activation). The results obtained from three independent experiments are shown. All data are expressed as mean ± SEM ((n =3). *p < 0.05, ****p < 0.0001 versus TNF-α alone.
